# First-Order Linear Mechatronics Model for Closed-Loop MEMS Disk Resonator Gyroscope

**DOI:** 10.3390/s20226455

**Published:** 2020-11-12

**Authors:** Hao Wang, Xiupu Wang, Jianbing Xie

**Affiliations:** MOE Key Laboratory of Micro and Nano Systems for Aerospace, Northwestern Polytechnical University, Xi’an 710072, China; wangh@mail.nwpu.edu.cn (H.W.); wang_xp@mail.nwpu.edu.cn (X.W.)

**Keywords:** MEMS, mechatronics model, order-reduction method (ORM), disk resonator gyroscope (DRG)

## Abstract

In this paper, a first-order closed-loop mechatronics model of a micro-electromechanical system (MEMS) disk resonator gyroscope (DRG) with a configurable ASIC is established for closed-loop design and performance analysis. There are usually some nonlinear modules in the gyroscope mechatronics model, and it is difficult to design the closed-loop controllers using classical automatic control theory. An order-reduction method (ORM) based on the Laplace transform and inverse Laplace transform is proposed to linearize the nonlinear modules. The linearized model is proved to show good agreement with the original mechatronics model in terms of system response. The experimental verification was conducted to demonstrate the validation of this method.

## 1. Introduction

The micro-electromechanical system (MEMS) disk resonator gyroscope (DRG) is a typical wine-glass mode gyroscope and it has become more favorable for high performance MEMS gyroscopes [1,2,3,4]. A lot of progress has been made on the disk resonator and DRG, including *Q* factor optimization [5,6,7,8], frequency tuning [9,10,11], resonator noise analysis and structure optimization [12,13,14,15], microcosmic dynamics analysis [16,17], nonlinear models of disk resonators [18,19], and fabrication processes [20,21]. 

Mostly, researchers are more concerned with accuracy or bias stability. Factors like bandwidth and closed-loop scale factor are usually ignored. The influence of closed-loop system and controller on detection bandwidth, settling time, and closed-loop gain, has not been discussed so far. For example, it is well believed that the bandwidth of a tuning-fork gyroscope is determined by the frequency difference of primary mode and secondary mode. For a central symmetrical gyroscope like DRG, however, the frequency difference is close to 0 after tuning, and the factors or the design method affecting the bandwidth have not been studied. Thus, it is of great significance to study how to determine these systematic parameters. These factors of a closed-loop gyroscopic system, such as bandwidth, closed-loop scale factor, settling time, could be determined by the classical automatic control theory. In an original closed-loop MEMS gyroscope system, there are typically some nonlinear modules, such as the modulator, demodulator, oscillator, and phase detector, making it difficult to design closed-loop controllers and analyze performance terms using classical automatic control theory. Therefore, it is quite necessary to linearize the mechatronics models in order to facilitate the closed-loop design [22,23,24,25,26,27]. 

In our previous work, we tried to model a vibrating ring gyroscope (VRG) [28]; however, it is mainly based on the force rebalance loop. In this paper, a mechatronics model is established for a MEMS disk resonator gyroscope (DRG) with a configurable ASIC (Application Specific Integrated Circuit). A model order-reduction method (ORM) based on Laplace transform and inverse Laplace transform is proposed to linearize the mechatronics model. Therefore, the gyroscopic model with a modulator and a demodulator in the AGC loop and rebalance loop is linearized to a first-order model, and the gyroscopic model with an oscillator and a phase detector in PLL is also linearized to a gain term. With the order-reduction model, the closed-loop controller and systematic terms can be easily designed using automatic control theory.

## 2. MEMS DRG and the Configurable ASIC

### 2.1. MEMS Disk Resonator

MEMS DRG is a typical wineglass mode resonator. A simplified model of a resonant vibratory gyroscope including equivalent noise force terms is given by [29]

(1) $$\begin{array}{l} m_{x}\frac{d^{2}x}{dt^{2}}+c_{x}\frac{dx}{dt}+k_{x}x=F_{elec}+2m_{y}\Omega_{z}\frac{dy}{dt}+F_{noise,x}(c_{x},t)\\ m_{y}\frac{d^{2}y}{dt^{2}}+c_{y}\frac{dy}{dt}+k_{y}y=-2m_{x}\Omega_{z}\frac{dx}{dt}+F_{noise,y}(c_{y},t) \end{array}$$

where *x*, *m_x_*, *c_x_*, and *k_x_* are the vibration displacement, effective mass, damping coefficient, and stiffness of the primary mode, respectively, and *y*, *m_y_*, *c_y_*, and *k_y_* are the vibration displacement, effective mass, damping coefficient, and stiffness of the secondary mode, respectively. *Ω_z_* is the angular rate of *Z*-axis. *F_noise_*_,*x*_ and *F_noise,y_* are mechanical noise of the primary mode and the secondary mode, respectively.

Ideally, it is assumed that the MEMS DRG is perfectly fabricated and the resonant frequencies, effective masses, and damping coefficient of the two modes are equal, i.e., *m_x_ = m_y_ = m_0_*, *k_x_ = k_y_ = k*, *c_x_ = c_y_ = c*. Then, the transfer functions of the two modes can both be expressed by

(2) G(s)=1m0s2+cs+k=1ms2+2ζωns+ωn2,

where *ω_n_* is the resonant frequency, ζ is the damping ratio, and $\zeta =\frac{c}{\sqrt{km_{0}}}$.

**Figure 1:**
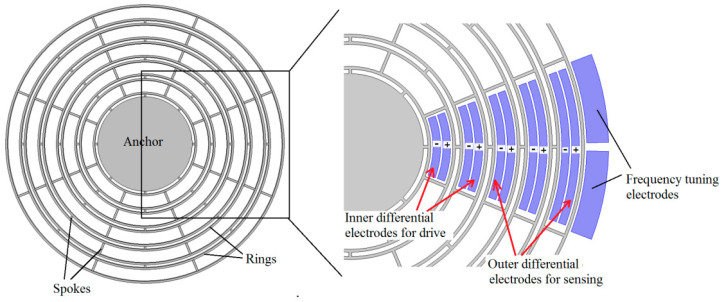
Diagrammatic sketch of MEMS disk resonator.

The DRG in this paper performs as n = 2 mode, as shown in Figure 1, and it enables differential driving and differential sensing. The diagrammatic sketch of the MEMS disk resonator and its electrodes involved in this paper is shown in Figure 1. The frequency split of the two modes can be tuned by the frequency tuning electrodes. The detailed parameters regarding DRG which will be used in later calculations are shown in Table 1.

### 2.2. A Configurable Closed-Loop ASIC for MEMS DRG

The closed-loop ASIC is an important device in achieving close-loop control. A configurable ASIC for MEMS gyroscopes is employed in this paper. The functions of the ASIC include providing high-voltage DC bias, temperature sensing and compensation, and frequency split tuning. However, the most important function is to realize closed-loop control for the MEMS gyroscope.

**Figure 2:**
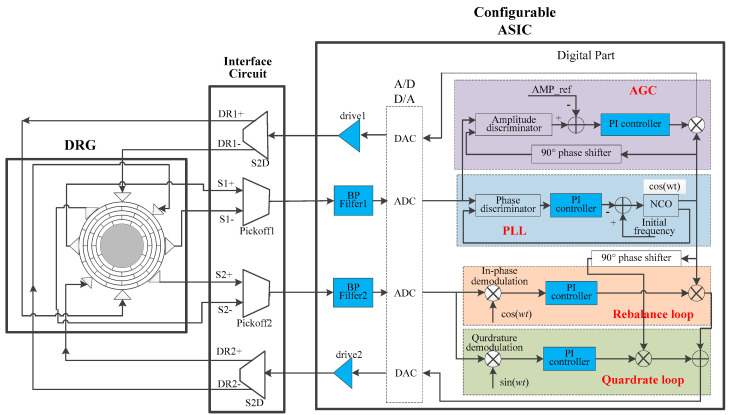
Closed-loop system constituted by the MEMS disk resonator and a configurable ASIC.

The architecture of the DRG closed-loop system is shown in Figure 2. The blue parts in the loops are configurable modules, which can be altered for performance terms design.

The analog parts of the ASIC mainly include analog band-pass filters, analog to digital converters (ADCs), digital to analog converters (DACs), and drive circuits. The digital parts of the ASIC can realize functions such as signal demodulation, signal modulation, PI controllers, and numerically controlled oscillator (NCO).

An interface circuit is needed between the resonator and ASIC. The interface circuit picks off the differential displacement signals and converts them to a single-end signal, and converts the single-end drive signal to differential drive signals.

**Figure 3:**
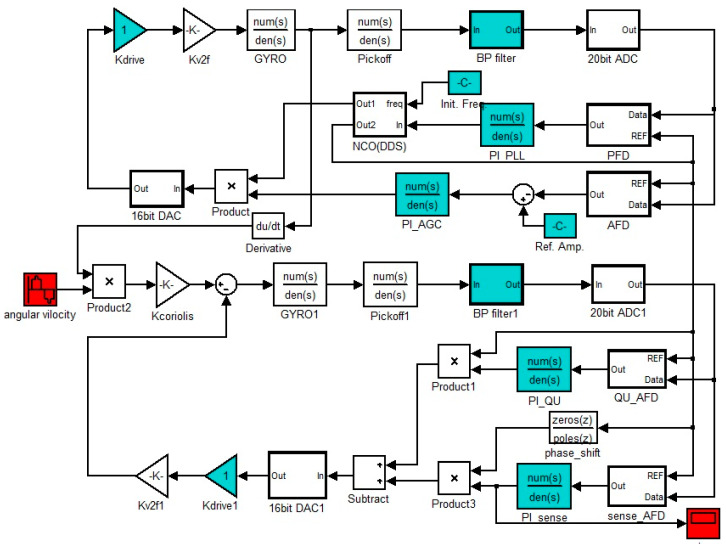
Original mechatronics model for DRG with a configurable ASIC.

An original mechatronic model is established on the Simulink platform as shown in Figure 3, which is consistent with the actual system consisting of the circuits and resonator.

The main modules involved in the model are introduced as follows.

(1) Pickoff circuit

**Figure 4:**
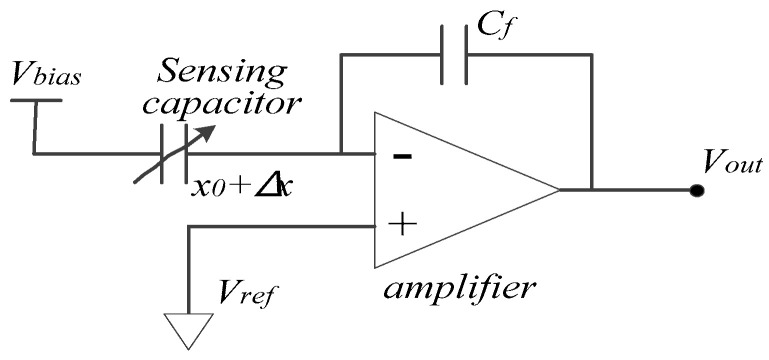
*C*/*V* circuit implemented by a charge amplifier.

The pickoff circuits mainly implement *C*/*V* (capacitance to voltage) detection. The *C*/*V* circuit employs a charge amplifier and its basic principle is shown in Figure 4.

The gain of the *C*/*V* circuit *k_CV_*, which represents the gain from displacement *x* to output voltage *V_out_*, is

(3) $$k_{CV}=\frac{\partial V_{out}}{\partial x}=\frac{V_{bias}C_{0}}{x_{0}C_{f}},$$

where *C*_0_ is the sensing capacitance, *V_bias_* is the DC bias voltage forced on proof mass, *x*_0_ is the spacing of electrodes’ gaps, and *C_f_* is the feedback capacitance.

(2) Analog band-pass filterPickoff circuit

The analog band-pass filter is implemented by a switched capacitor circuit, which mainly filters the white noise and 1/f noise from previous stage circuits. The transfer function of the band-pass filter is represented by *H_BPF_*(*s*). When the gyroscope resonator vibrates near the resonant frequency, the band-pass filter can be seen as a gain term for the effective signal, denoted by *k_BPF_*. The analog band-pass filter is a configurable part whose center frequency and gain can be configured to accommodate different resonators.

(3) ADCs and DACs

The ASIC employs 20-bit sigma-delta ADCs and 16-bit sigma-delta DACs. The gain of ADC is expressed by *k_ADC_* and the gain of DAC is expressed by *k_DAC_*.

(4) Drive circuit

The drive circuit provides the drive capability for DRG. The gain of drive circuit is configurable, and it is expressed by *k_drive_*.

(5) Digital circuit

The digital circuit undertakes the main operations of the closed-loop control system. For the primary mode, the main functions of the digital circuit include digital phase detection, digital demodulation, digital PI controllers, NCO, etc. For the secondary mode, the main operations of the digital circuit include orthogonal demodulation, in-phase demodulation, PI controllers, modulation (digital multiplier), etc.

(6) Gain of electrostatic feedback force

A high DC bias voltage *V_bias_* is applied on the mass and the feedback drive voltage *V_drive_* is applied on drive electrodes. Thus, the electrostatic feedback force *F_elec_* is expressed by

(4) $$F_{\mathrm{elec}}=\frac{1}{2}\times \frac{V_{bias}C_{drive}V_{drive}}{x_{0}}.$$

Additionally, the gain of the electrostatic feedback force *k_V2F_* is

(5) $$k_{V2F}=\frac{1}{2}\times \frac{V_{bias}C_{drive}}{x_{0}},$$

where *C_drive_* is the drive capacitance.

(7) Gain of Coriolis force

According to the Coriolis Effect [30], the Coriolis force is

(6) $$F_{coriolis}=-4A_{g}m_{0}v_{x}\times \Omega ,$$

where *Ag* is angular gain, *v_x_* is vibrating velocity of the primary mode, and

(7) $$v_{x}=\frac{d(A_{x}\sin(\omega_{0}t))}{dt}=A_{x}\omega_{0}\times \cos(\omega_{0}t),$$

where *cos*(*ω*_0_*t*) acts as a modulation term and *A_x_* is amplitude of the primary mode. Thus, the Coriolis gain is

(8) $$k_{coriolis}=-4A_{g}m_{0}A_{x}\omega_{0}.$$

The specifications of ASIC and controller are presented in Table 2.

## 3. Model Linearizing and PI Controllers’ Design for Primary Mode

### 3.1. AGC Loop Model Order-Reduction and Closed-Loop Design

The AGC loop has frequency-shifting modules, including a modulator and a demodulator, which make the design of a closed-loop system complicated. To design a closed-loop control system using classical automatic control theory, it is necessary to simplify the closed-loop system to obtain a linear system. For this purpose, the order-reduction method (ORM) is proposed in this paper. With the ORM, the system response of a complex high-order system composed of multiple nonlinear modules and a gyroscopic module in series can be derived. The complex high-order system in series can be replaced by a low-order linear system whose system response is equivalent or approximate to the complex high-order system to achieve a linearizing system.

**Figure 5:**
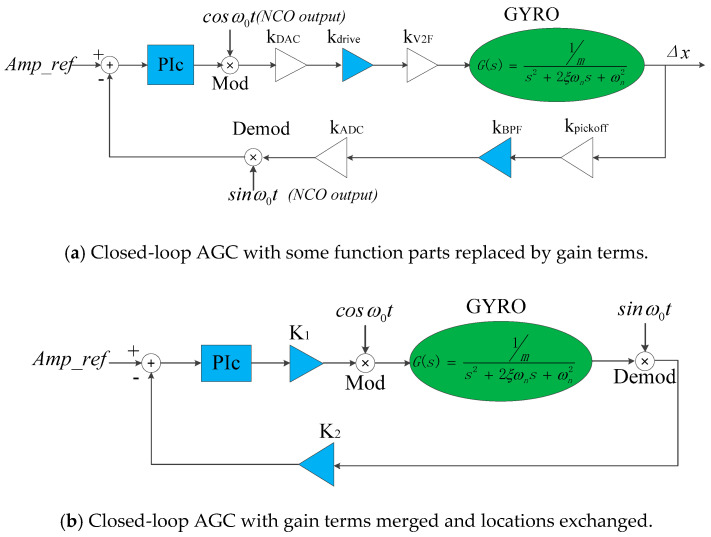
Simplification process of closed-loop AGC.

First, the modules that do not change the frequency of effective signals are replaced by gain terms as shown in Figure 5a. This is called the “equivalent gain method”. Then, the gain terms are merged as *K*_1_ and *K*_2_, and $K_{1}=k_{DAC}\times k_{drive1}\times k_{V2F}$, $K_{2}=k_{pickoff}\times k_{BPF}\times k_{ADC}$. The locations of some gain terms are exchanged because this does not change the closed-loop characteristics, as shown in Figure 5b. The term *Amp_ref* is the amplitude control term of primary mode in the digital circuit.

Second, the controlled objects of the closed-loop are the modules <modulator–gyroscope–demodulator> in series instead of gyroscope according to Figure 5b, thus, the ORM is adopted and the modules <modulator–gyroscope–demodulator> in series are derived as a first-order transfer function *G*_1_(*s*):

(9) G1(s)=12mωns+ζωn.

The derivation process for Equation (9) is given below.

**Figure 6:**
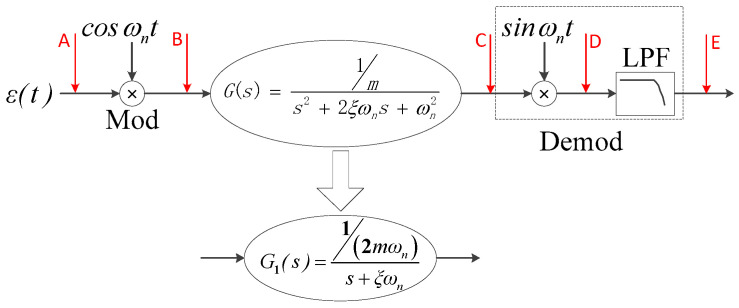
The modules <modulator–gyroscope–demodulator> in series and their equivalent first-order transfer function.

The signal representations of the different nodes are given in Figure 6. The input signal at point *A* is set to be the unit step signal *ε*(*t*). Then, the signal at point *B* equals the unit step signal multiplied by the modulation signal *cos*(*ω_n_t*), i.e.,

(10) $$Y_{B}(t)=\varepsilon (t)\times \cos\omega_{n}t.$$

A Laplace transformation is performed on *Y_B_*(*t*):

(11) $$H_{B}(s)=L(Y_{B}(t))=\frac{s}{s^{2}+\omega_{n}^{2}}.$$

After the signal passes through the gyroscopic second-order system and reaches point *C*, the transfer function from point *A* to point *C* is

(12) $$\begin{array}{l} H_{C}(s)=H_{B}(s)\times G(s)\\ =\frac{1}{2\zeta \omega_{n}^{2}m}\times [\frac{\omega_{n}}{s^{2}+\omega_{n}^{2}}-\frac{\omega_{n}}{s^{2}+2\zeta \omega_{n}s+\omega_{n}^{2}}] \end{array}$$

An inverse Laplace transformation is performed on *H_C_*(*s*) to obtain a time domain signal at point *C*:

(13) $$\begin{array}{l} Y_{C}(t)=L^{-1}(H_{C}(s))\\ =\frac{1}{2\zeta \omega_{n}^{2}m}\times [\sin\omega_{n}t-\frac{1}{\sqrt{1-\zeta^{2}}}e^{-\zeta \omega_{n}t}\sin(\omega_{n}\sqrt{1-\zeta^{2}}t)] \end{array}$$

For vacuum encapsulation gyroscopes, the *Q* factor is usually very large and Q>10,000, so $\zeta =\frac{1}{2Q}\to 0$ and $\sqrt{1-\zeta^{2}}\approx 1$. Then:

(14) $$Y_{C}(t)=\frac{\sin\omega_{n}t}{2\zeta \omega_{n}^{2}m}\times (1-e^{-\zeta \omega_{n}t}).$$

In the demodulation phase, the signal at point *C* is multiplied by the demodulation signal *sin*(*ω_n_t*) to obtain a signal at point *D*:

(15) $$\begin{array}{l} Y_{D}(t)=Y_{C}(t)\times \sin\omega_{n}t\\ =\frac{1}{2\zeta \omega_{n}^{2}m}\times (1-e^{-\zeta \omega_{n}t})\times \frac{1}{2}(1-\cos2\omega_{n}t) \end{array}$$

The signal at point *D* passes through a low-pass filter in the demodulation module and reaches point *E*, and the high-frequency component $\omega =2\omega_{n}$ is filtered out so that only the signal envelope is obtained:

(16) $$Y_{E}(t)=\frac{1}{2\zeta \omega_{n}^{2}m}\times (1-e^{-\zeta \omega_{n}t}).$$

*Y_E_(t)* is the signal that the unit step signal transmits from point *A* to point *E*; that is, *Y_E_(t)* is the unit step response of the modules <modulator–gyroscope–demodulator> in series.

An inverse Laplace transformation is performed on *Y_E_(t)*:

(17) $$H_{E}(s)=L^{-1}(Y_{E}(t))=\frac{1}{2\omega_{n}m}\times (\frac{1}{s(s+\zeta \omega_{n})}).$$

It is assumed that the equivalent transfer function of the modules <modulator–gyroscope–demodulator> in series is *G*_1_*(s)* and

(18) $$G_{1}(s)=\frac{H_{E}(s)}{R(s)},$$

where *R(s)* is the Laplace transformation of the step signal *ε(t)* and $R(s)=L(\varepsilon (t))=\frac{1}{s}$. Thus:

(19) G1(s)=12mωns+ζωn.

**Figure 7:**
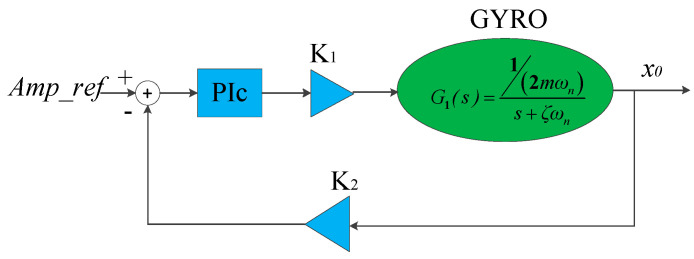
Order-reduction model for AGC loop.

Then the AGC loop is linearized to the model shown in Figure 7. After model order-reduction, the closed-loop transfer function of the AGC loop can be expressed by

(20) $$C_{AGC}(s)=\frac{K_{1}\times PI(s)\times G_{1}(s)}{K_{1}\times PI(s)\times G_{1}(s)\times K_{2}+1}.$$

Here, *PI(s)* represents the transfer function of the PI controller. It is convenient to design the PI controller based on Equation (20). A controller design method based on time domain characteristics is adopted. The structure of the PI controller is

(21) $$PI(s)=\frac{b(s+\zeta \omega_{n})}{s(s+a)}.$$

where the terms *a* and *b* are the parameters of the PI controller.

Thus, the closed-loop transfer function can be expressed by

(22) CAGC(s)=ωnc2K2s2+2ζcωncs+ωnc2=K1×bmωns2+as+K1×K2×bmωn,

where the terms *ζ_c_* and *ω_nc_* are the damping ratio and resonant frequency, respectively, of the second-order closed-loop system *C_AGC_*(*s*) and, $2\zeta_{c}\omega_{nc}=a$, ωnc=K1×K2×bmωn.

To avoid overshoot of the system, the systematic damping ratio *ζ_c_* is set to 1; that is, the AGC closed-loop system is a critical damping system. The term ζ_c_*ω_nc_ determines the systematic bandwidth and the rise time of step response. According to the automatic control theory [31], when *ζ_c_* = 1, the bandwidth *Bw* is $Bw=0.64\omega_{nc}$ (rad/s) and the rise time of step response is $Tr=\frac{5.84}{\omega_{nc}}(s)$. 

According to the configuring of ASIC and parameters of DRG, $K_{1}=1.36\times 10^{-9}$ and $K_{2}=3.78\times 10^{11}$.

When the system bandwidth of the AGC loop is set to 10 Hz, the rise time is 0.06 s, and the PI controller is $PI(s)=\frac{5.432\times (s+3.232)}{s(s+200)}$; therefore, the closed-loop transfer function is $C(s)=\frac{2.6455\times 10^{-8}}{s^{2}+200s+1\times 10^{4}}$. When the digital amplitude control term *Amp_ref* is set to a constant, the amplitude of primary mode *A_x_* is

(23) $$\frac{A_{x}}{Amp\_ref}=C(s){|}_{j\omega =0}=\frac{1}{K_{2}},$$

**Figure 8:**
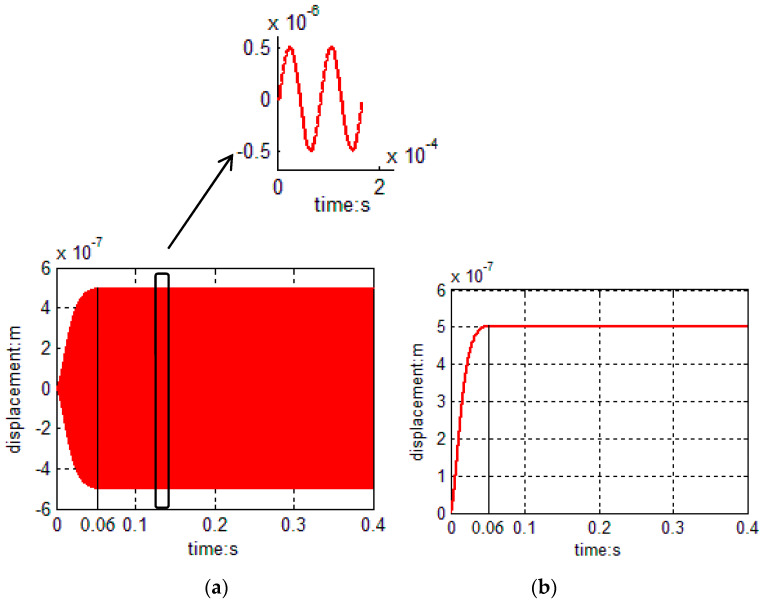
(**a**) Step response of AGC original model and (**b**) step response of the AGC linearized model.

The amplitude of primary mode is set to 0.5 μm; therefore, $Amp\_ref=1.89\times 10^{5}$. The PI controller is introduced to the original model and the linearized model, respectively. The step response of the original model is shown in Figure 8a and the step response of the linearized model is shown in Figure 8b. 

According to Figure 8, the rise times of both models are 0.06 s, which are subordinated to designed values, and there is no overshot in both models. The simulation results show that the step response of the linearized model is completely consistent with the envelope of the original model’s step response. 

**Figure 9:**
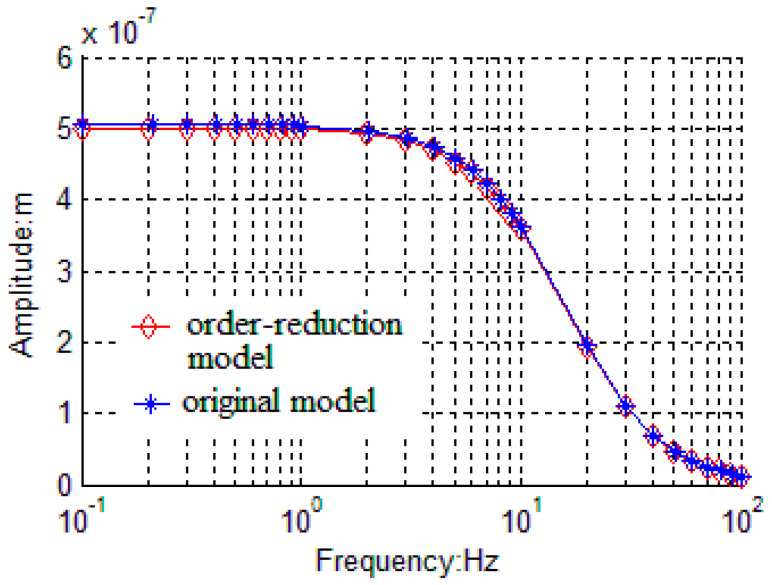
Frequency response characteristics of AGC loop based on the linearized model and the original model.

The frequency response characteristics are also simulated based on both the linearized model and the original model as shown in Figure 9. The amplitude of the gyroscope in order-reduction model is 5 × 10^−7^ m while amplitude in original model is 5.07 × 10^−7^ m. The amplitude error is 1.4% between the two models. Additionally, 3dB bandwidths of the two models are both 10 Hz, which are quite consistent with designed bandwidth. Thus, the frequency responses of the two models are almost identical. 

**Figure 10:**
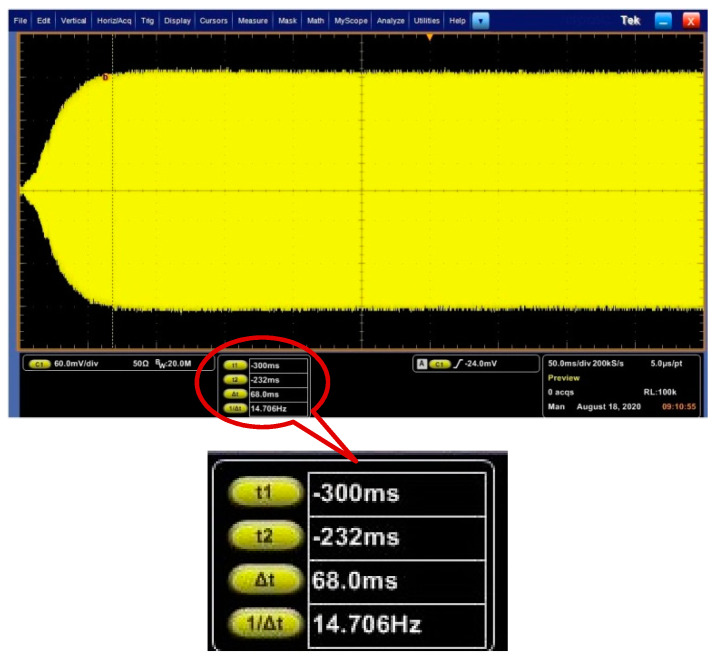
Start-up waveform of AGC loop with DRG experimental board.

The experimental verification was conducted with a DRG test board. The designed PI controller of AGC loop is implemented in the ASIC. When the output frequency of NCO is fixed at the resonance frequency, the *C*/*V* output of primary mode which can represent displacement amplitude of primary mode, is tested using an oscilloscope, as shown in Figure 10. The rise time of the amplitude is 68 ms. The error of rise time between the experiment and model is about 13%, which is acceptable. Therefore, the experiment verified the validation of the linearization method of the AGC loop.

According to the frequency responses and the step responses of the two models, it can be concluded that the linearized model is interchangeable with the original model. Therefore, closed-loop performance analysis and closed-loop controller design can be performed based on the linearized model.

### 3.2. PLL Model Linearizing and Closed-Loop Design 

**Figure 11:**
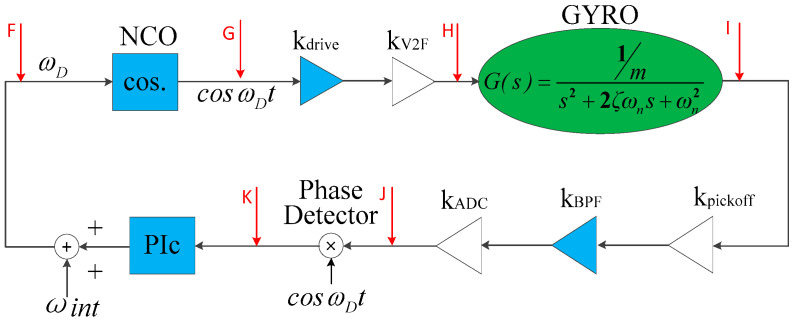
PLL loop after using equivalent gain method.

The PLL model mainly contains a digital phase detector, a PI controller, and a NCO. The NCO formed by a DDS (Direct Digital frequency Synthesizer) generates a sinusoidal or cosine signal. At the input of the NCO, an initial frequency *ω_int_*, which is close to the resonant frequency, is generally added to reduce the time of the phase-lock and improve the efficiency of the phase-locked loop. The equivalent gain method is first used to simplify the PLL to the loop structure shown in Figure 11. At time *t*, it is assumed that the frequency of point *F* is *ω_D_* and the output of the NCO at point *G* is $Y_{G}\left(t\right)=\cos\left(\omega_{D}t\right)$. Then, the signal at point *H* is $Y_{H}\left(t\right)=K_{3}\cos\left(\omega_{D}t\right)$; here, $K_{3}=k_{\mathrm{drive}}\times k_{V2F}$.

According to the amplitude-frequency characteristics of the gyroscopic resonator, the signal that reaches point *I* through the gyroscopic resonator is

(24) $$Y_{I}\left(t\right)=K_{3}*A\left(\omega_{D}\right)\cos\left(\omega_{D}t+\theta \right)$$

where

(25) A(ωD)=1mωn2[1−(ωDωn)2]2+(2ζωDωn)2 and

(26) $$\theta =p(\omega_{D})=\arctan(\frac{-\frac{2\zeta \omega_{D}}{\omega_{n}}}{1-{\left(\frac{\omega_{D}}{\omega_{n}}\right)}^{2}}).$$

The input of the phase detector is set to Δ*ω* (Δ*ω = ω_n_ − ω_D_*); then, *θ* can be expressed by

(27) $$\theta =p(\Delta \omega )=\arctan(\frac{\omega_{n}(\omega_{n}-\Delta \omega )}{(2\omega_{n}-\Delta \omega )\Delta \omega }).$$

The signal at point *J* is

(28) $$Y_{J}(t)=K_{3}K_{4}\times A(\omega_{D})\cos(\omega_{D}t+\theta ),$$

where $K_{4}=k_{pickoff}\times k_{BPF}\times k_{ADC}$.

The signal reaching point *K* is

(29) $$\begin{array}{l} Y_{K}(t)=K_{3}K_{4}A(\omega_{D})\cos(\omega_{D}t+\theta )\times \cos(\omega_{D}t)\\ =K_{3}K_{4}A(\omega_{D})[\cos(2\omega_{D}t+\theta )+\cos\theta ] \end{array}$$

As the phase detector has a low-pass filter function, the signal component at the frequency of *2ω_D_* is filtered out. Therefore, the signal at point *K* is

(30) $$Y_{K}(t)=K_{3}K_{4}A(\omega_{D})\cos\theta .$$

**Figure 12:**
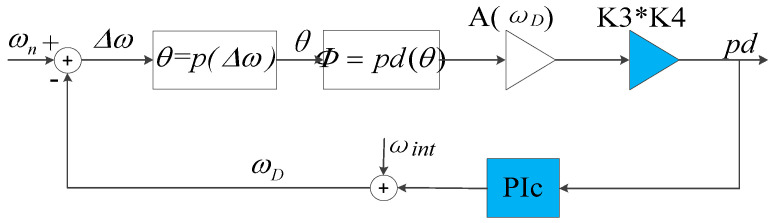
Order-reduction model of PLL.

Thus, the PLL shown in Figure 11 can be further derived to the loop shown in Figure 12.

In the loop shown in Figure 12, θ=p(Δω) and Φ=cos(θ) are both nonlinear modules. It is not convenient to design the closed-loop controller with automatic control theory; therefore, it is necessary to approximate the two modules to obtain a linear system. 

**Figure 13:**
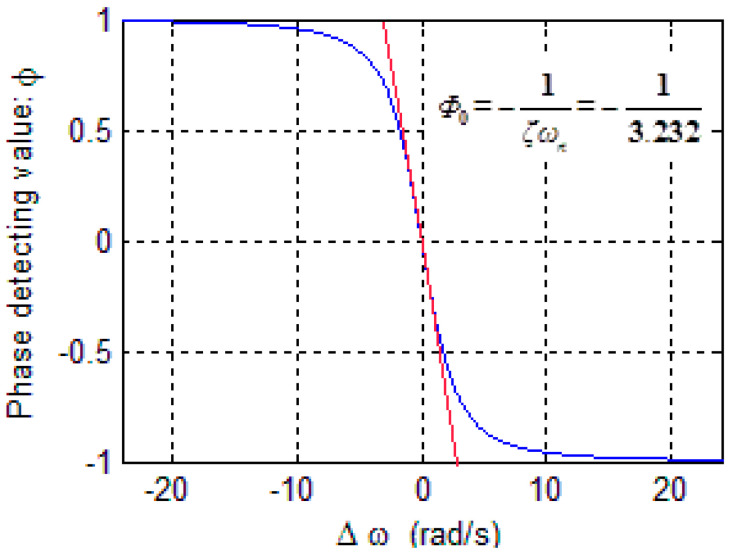
Relationship curve between *Φ* and Δω.

According to Equations (27) and (30), the relationship between *Φ* and Δ*ω* is plotted as shown in Figure 13. The slope of the curve in Figure 13 represents the gain from Δ*ω* to *Φ*; however, the slope varies with Δ*ω*. When PLL is stable, Δ*ω = 0*; thus, the slope at Δ*ω = 0* is used as the gain from Δ*ω* to *Φ* while designing the loop controller.

When the frequency of the drive signal *ω_D_* approaches the gyroscopic resonant frequency *ω_n_*, A(ωD)≈12ζmωn2=Qk and θ≈90°. When Δ*ω*→0, the slope of the curve is expressed by

(31) $$\Phi_{0}=\frac{d\Phi }{d(\Delta \omega )}|{}_{\Delta \omega \to 0}=-\frac{1}{\zeta \omega_{n}}.$$

Thus, when the PLL is stable, that is Δ*ω*→0, the modules <oscillator–gyroscope–phase detector> in series in PLL can be equivalent to a gain term:

(32) $$pd(\Delta \omega )=-\frac{1}{\zeta \omega_{n}}\times A(\omega_{n}).$$

**Figure 14:**
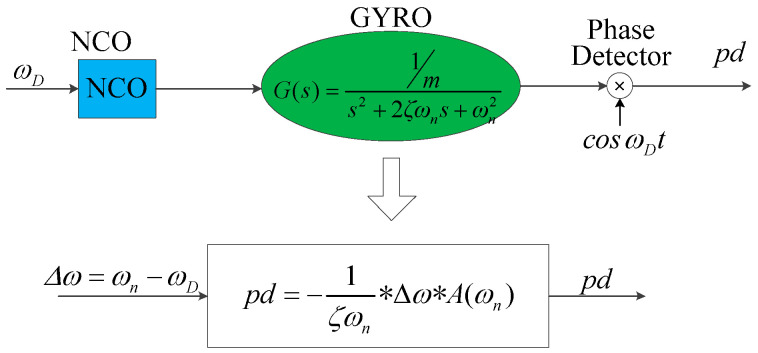
The models < oscillator–gyroscope–phase detector > in series are approximated as a gain term.

**Figure 15:**
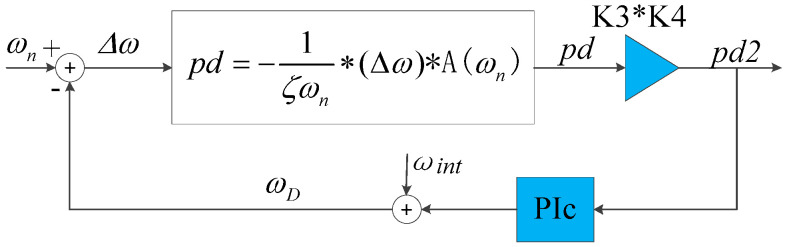
Linearized PLL.

Here, the gain term in Equation (32) could be considered as the equivalent gain when *ω_D_* does not equal to *ω_n_*. Therefore, the models <oscillator–gyroscope–phase detector> in series can be approximated as a gain term as shown in Figure 14 when designing the closed-loop controller. The PLL can be linearized as shown in Figure 15. 

The closed-loop controller is designed based on the linearized PLL loop. The PI controller is determined as $PI_{PLL}(s)=\frac{b}{s(s+a)}$; then, the closed-loop transfer function of linearized PLL is

(33) $$\begin{array}{l} C_{PLL}(s)=\frac{\omega_{nc}^{2}}{s^{2}+2\zeta_{c}\omega_{nc}s+\omega_{nc}^{2}}\\ =\frac{K_{1}K_{2}A(\omega_{D})\Phi_{0}\times b}{s^{2}+as+K_{1}K_{2}A(\omega_{D})\Phi_{0}\times b} \end{array}$$

Here, $2\zeta_{c}\omega_{nc}=a$ and $\omega_{nc}=\sqrt{K_{1}K_{2}A(\omega_{D})\Phi_{0}\times b}$.

According to the configuring of ASIC and parameters of DRG, $K_{3}=3.44\times 10^{-7}$, $K_{4}=3.78\times 10^{11}$, and $A(\omega_{D})=1.108$.

The systematic damping ratio of PLL *ζ_c_* is designed to be 6 and for over-damping systems, the rise time (90% of the maximum value) is expressed by [31] $Tr=\frac{1+1.5\zeta_{c}+\zeta_{c}{}^{2}}{\omega_{nc}}$. The rise time is designed as 0.2 s, so $\omega_{nc}\approx 300$. According to Equation (33), the PI controller of PLL is:

(34) $$PI_{PLL}(s)=\frac{0.021}{s(s+3600)}.$$

**Figure 16:**
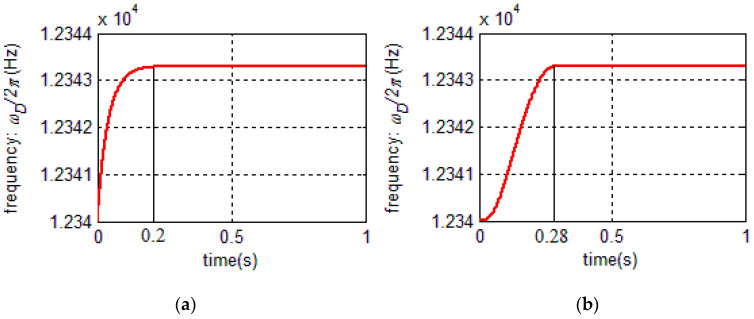
(**a**) Step response of the PLL linearized model and (**b**) step response of the PLL original model.

The initial frequency *ω_int_* is set to $\omega_{\mathrm{int}}=12340*2\pi$ and the PI controller is introduced to the linearized model and the original model, respectively. The step response curves of the two models are shown in Figure 16a,b, respectively. According to Figure 16, the step response of the linear PLL model differs a little from the original model.

The rise time of the original model is 0.28 s while the rise time of the ideal model is 0.2 s. The rise time of the original model is longer than that of the linear model. This is because the equivalent gain term in linearized PLL is an approximation. Actually, the gain in the original PLL model is time-varying and is smaller than the equivalent gain term in linearized PLL when the PLL system is unstable. Therefore, the rise time of original PLL model is longer than that of the linear model, the linear PLL model is not completely maintained with the original model in the quantitative design. However, it is still of great significance for the qualitative analysis for the original model.

**Figure 17:**
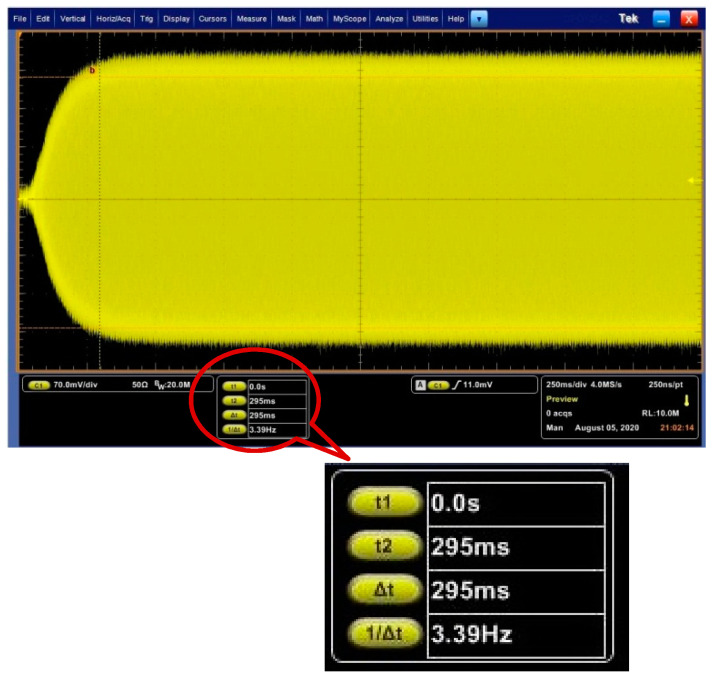
Start-up waveform of PLL loop with DRG experimental board.

The experimental verification of PLL loop is also conducted with the designed PI controller. As the frequency cannot be directly reflected, and for PLL loop, only when the frequency is stable, the amplitude is stable, we consider the stable time of amplitude as the frequency stable time. We tested the start-up amplitude of primary mode when the AGC loop does not work. The waveform is shown in Figure 17. The stable time of PLL loop is 295 ms which is close to the rise time of original model, and the error is about 5%. 

So far, the gyroscopic model with a modulator and a demodulator in AGC loop is linearized to a first-order model, and the gyroscopic model with an oscillator and a phase detector in PLL is also linearized to a gain term. The PI controllers of the two loops are both designed using classical automatic control theory based on linearized models. The designed PI controllers are introduced to the original model as well as DRG experimental board, and it is verified that the method to linearize the gyroscopic model is useful. 

## 4. Model Linearizing and Performance Analysis of Rebalance Loop

### 4.1. Model Order-Reduction and Closed-Loop Design of Rebalance Loop

The secondary mode includes a rebalance loop and a quardrate loop. As the basic principles and models of the two loops are almost the same and the rebalance loop determines many main performance terms of gyroscope, the rebalance loop is mainly analyzed.

**Figure 18:**
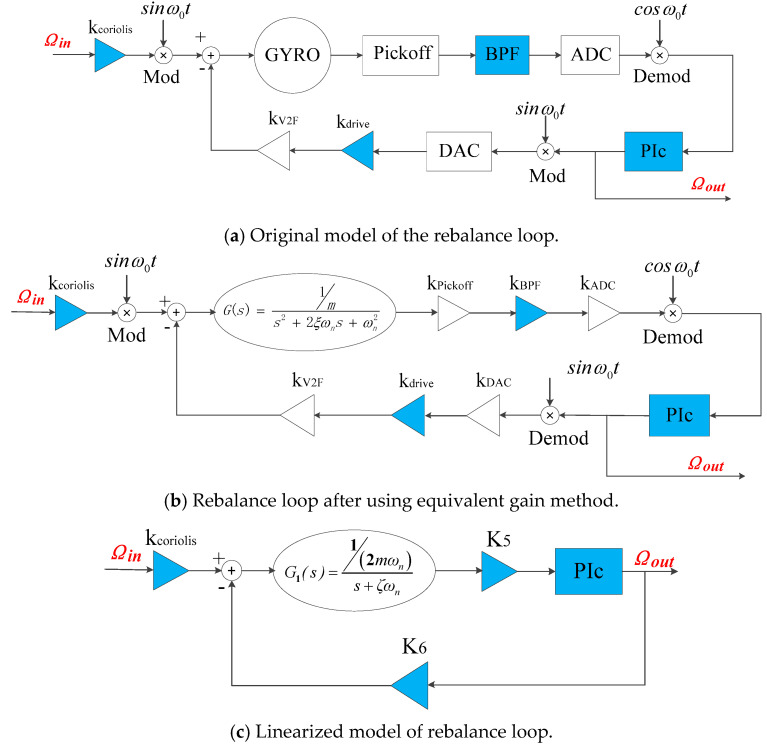
Linearization process of the rebalance loop.

The rebalance loop in the original model is shown in Figure 18a. The equivalent gain method is used to simplify the rebalance loop to the loop structure as shown in Figure 18b.

According to the conclusion in Section 3.1, the rebalance loop is order-reduced to a simplified loop as shown in Figure 18c. The terms *K*_5_ and *K*_6_ in Figure 18c are:

(35) $$K_{5}=k_{CV}\times k_{pickoff}\times k_{BPF}\times k_{ADC},\;\mathrm{and}$$

(36) $$K_{6}=k_{V2F}\times k_{\mathrm{dri}ve}\times k_{DAC}\sqrt{2}.$$

The closed-loop transfer function of the rebalance loop is

(37) $$C_{RB}(s)=k_{coriolis}\times \frac{K_{5}\times PI(s)\times G_{1}(s)}{K_{5}\times K_{6}\times PI(s)\times G_{1}(s)+1}.$$

The PI controller of the rebalance loop is determined by $PI(s)=\frac{b(s+\zeta \omega_{n})}{s(s+a)}$; then, the closed-loop transfer function turns to

(38) CRB(s)=kcoriolis×ωnc2K6s2+2ζcωncs+ωnc2=kcoriolis×K5×bmωns2+as+K5×K6×bmωn

where the terms *ζ_c_* and *ω_nc_* are the systematic damping ratio and resonant frequency of *C_RB_(s)*, respectively, and $2\zeta_{c}\omega_{nc}=a$, ωnc=K5×K6×bmωn.

### 4.2. Bandwidth Analysis

According to the closed-loop transfer function shown in Equation (38), the bandwidth can be designed by configuring the terms *ζ_c_* and *ω_nc_*. If ζc is set to 1, then the bandwidth *Bw* is $Bw=0.64\omega_{nc}$.

Here, the bandwidth is desired to 50 Hz, then $\omega_{nc}=490$. The terms *K*_5_ and *K*_6_ are calculated as $K_{5}=1.64\times 10^{14}$ and $K_{6}=1.36\times 10^{-11}$. Then the parameters of PI controller *a* and *b* are a=980 and b=27.95. Therefore, the closed-loop transfer function of the rebalance loop is

(39) $$C_{RB}(s)=\frac{1.797\times 10^{9}}{s^{2}+980s+2.401\times 10^{5}}.$$

**Figure 19:**
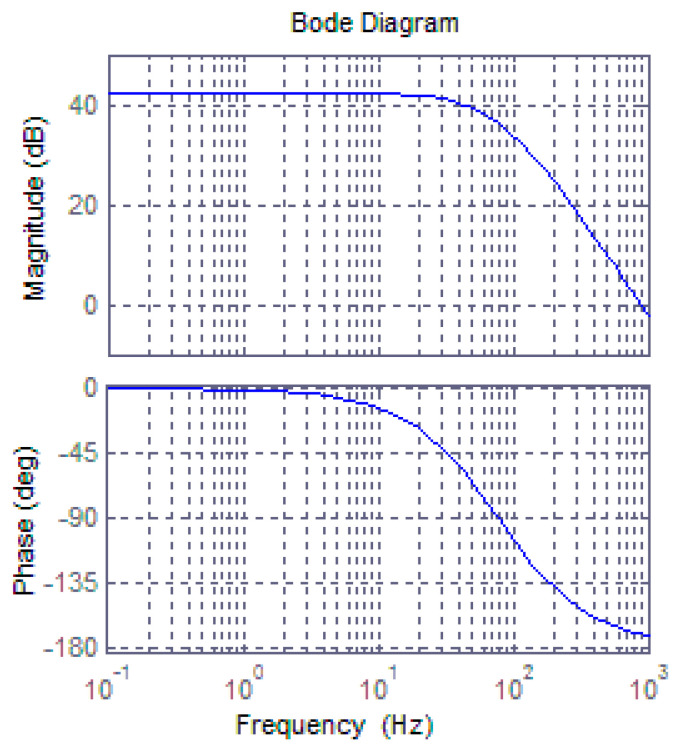
Bode diagram of the rebalance loop’s linearized model.

The Bode diagram of the closed-loop transfer function, which represents the frequency response of linearized model, is shown in Figure 19.

**Figure 20:**
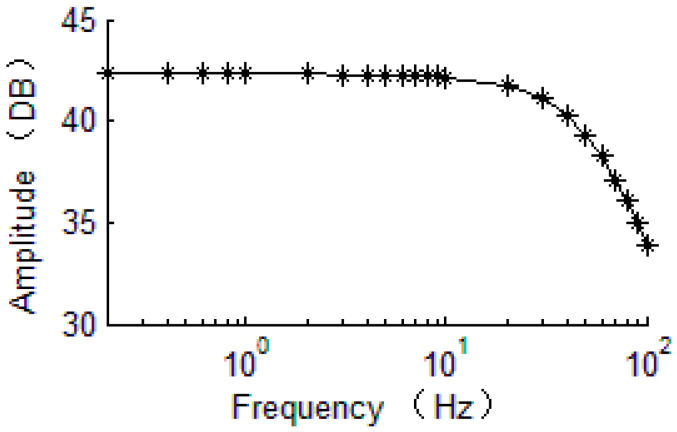
Amplitude–frequency characteristic curve of the original model.

The frequency response of the original rebalance loop is simulated with the designed PI controller as shown in Figure 20 and the amplitude–frequency characteristic curve shows accordance with that of the linearized model. Therefore, the bandwidth design based on the linearized model is effective for the original model.

**Figure 21:**
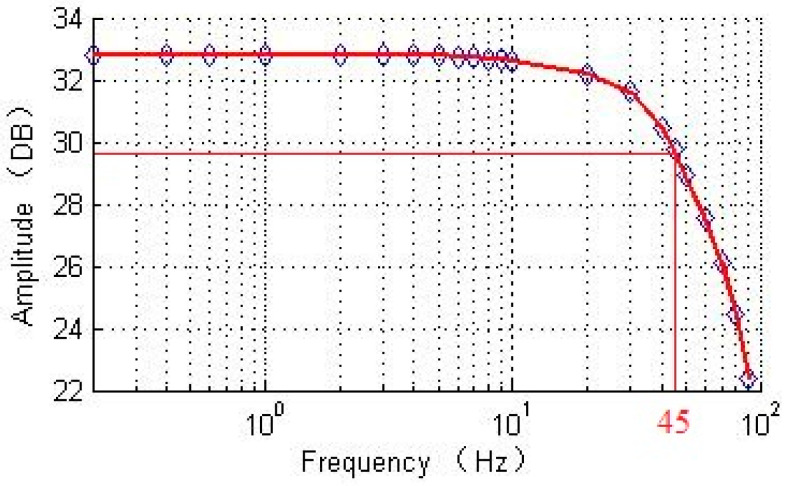
Tested bandwidth of DRG experimental board.

The bandwidth of the DRG test system was carried out. The test result is shown in Figure 21. The tested bandwidth is 45 Hz. Compared with the designed bandwidth of the linear model, the error is 11%, which is acceptable. The experiment illustrates that the PI controller based on linear model is suitable for the real gyroscopic system.

## 5. Discussion

In this paper, a closed-loop mechatronics model of a MEMS DRG with a configurable ASIC was established. The mechatronics model was linearized by the proposed order-reduction method (ORM). The controllers of all loops were designed using classical automatic control theory based on the linearized mechatronics. The linearized model was proved to show good agreement with the original mechatronics models in terms of system response. Meanwhile, the experimental verification was also carried out to demonstrate the validation of the method.

Any complex system needs to be reduced to an ideal system in order to use theoretical knowledge for analysis. Our work has built a bridge between the original mechatronics model and an ideal model. With the ideal model, the closed-loop system controller can be easily designed using classical automatic control theory and the systematical factors such as start-up time and bandwidth can be mathematically analyzed, which could hardly be done with the original mechatronics model. Depending on these analyses and mathematical conclusion, it is also helpful to guide the design of gyroscope resonator as well as circuit.

## Figures and Tables

**Table 1:** Key parameters of disk resonator and its sensing system.

Parameter	Value
Effective mass/*m*	1.67 × 10^−6^ kg
*Q* factor/*Q*	13,000
Resonant frequency/*f*_0_	12,343.3 Hz
Angular gain/*A_g_*	0.393
Sense capacitance/*C*_0_	7.6 pF
Drive capacitance/*C_drive_*	3.4 pF
Electrodes’ spacing/*x*_0_	5 μm
Primary amplitude/*A_x_*	0.5 μm
Feedback capacitance of *CV*/*C_f_*	10 pF
DC bias voltage/*V_bias_*	10 V

**Table 2:** Key parameters of disk resonator.

Key Parts of ASIC	Specification	Value
Pickoff circuit/CV	To pickoff the displacement of resonator	$k_{CV}=\frac{\partial V_{out}}{\partial x}=\frac{V_{bias}C_{0}}{x_{0}C_{f}}$
DAC/ADC	20-bit sigma-delta ADCs and 16-bit sigma-delta DACs	$k_{ADC}=2^{20}$, $k_{DAC}=2^{-16}$
Band-pass filter	The central frequency is configurable	$H_{BPF}(s)=\frac{3.3\times 10^{5}s}{s^{2}+4.01s+5.42\times 10^{9}}$
Drive circuit	To provide the drive capability for DRG	Gain is configurable
Electrostatic feedback force	-	$k_{V2F}=\frac{1}{2}\times \frac{V_{bias}C_{drive}}{x_{0}}$
Gain of Coriolis force	-	$k_{coriolis}=-4A_{g}m_{0}A_{x}\omega_{0}$
PI controller	Implemented in an embedded MCU and is configurable	$PI(s)=\frac{b(s+\zeta \omega_{n})}{s(s+a)}$, configurable
